# Plasma microRNAs to Select Optimal Patients for Antibody Production from Anti-Addiction Vaccines

**DOI:** 10.3390/vaccines13020181

**Published:** 2025-02-13

**Authors:** Thomas R. Kosten, Amrit Koirala, David A. Nielsen, Coreen B. Domingo, Ynhi T. Thomas, Preethi H. Gunaratne, Cristian Coarfa

**Affiliations:** 1 Department of Psychiatry and Behavioral Sciences, Department of Pharmacology, Department of Neuroscience, Department of Immunology, Baylor College of Medicine, Houston, TX 77030, USA; 2 Center for Precision Environmental Health, Baylor College of Medicine, Houston, TX 77030, USA; amrit.koirala@bcm.edu; 3 Department of Psychiatry and Behavioral Sciences, Baylor College of Medicine, Houston, TX 77030, USA; isailfast@gmail.com (D.A.N.); cdomingo@bcm.edu (C.B.D.); 4 Henry J.N. Taub Hospital Department of Emergency Medicine, Baylor College of Medicine, Houston, TX 77030, USA; ynhi.thomas@bcm.edu; 5 Michael E. DeBakey VA Medical Center, Center for Innovations in Quality, Effectiveness, and Safety, Houston, TX 77030, USA; 6 Department of Biology and Biochemistry, University of Houston, Houston, TX 77204, USA; phgunara@central.uh.edu; 7 Dan L. Duncan Comprehensive Cancer Center, Baylor College of Medicine, Houston, TX 77030, USA; coarfa@bcm.edu

**Keywords:** human vaccine, cocaine, microRNA, antibody response, epigenetics, drug overdose, biomarker

## Abstract

**Background**: Cocaine and illicit amphetamines (disguised as “Adderall”) are being laced with fentanyl and producing accidental and intentional fatal overdoses. Vaccines can prevent these overdoses, but 33% of humans generate insufficient anti-drug antibody (AB) levels. Plasma microRNAs (miRs) can be used to predict non-responders. We have plasma stored from 152 cocaine vaccine trial participants following three vaccinations over 9 weeks and examined miRs as potential response biomarkers. **Methods**: We compared 2517 miRs before anti-cocaine vaccination in participants with the highest (n = 25) to the lowest (n = 23) antibody levels. False Discovery Rates (FDRs) were applied to identify differentially expressed (DE) miRs. We used miR target prediction pipelines to identify the miR-regulated genes. **Results**: Using a DE-FDR < 0.05 and a >3-fold difference between high- and low-AB responders yielded 12 miRs down and 3 miRs up compared to low-AB patients. Furthermore, 11 among 1673 genes were targeted by 3 or more of the 12 down DE-miRs. **Conclusions**: A significant DE-miR for identifying optimal antibody responders replicated previous vaccine study predictors (miR-150), and several more miRs appear to be strong candidates for future consideration in replications based upon significance of individual DE-miRs and upon multiple miRs converging on individual genes.

## 1. Introduction

The fentanyl overdose epidemic has significantly increased fatalities from both opioid and, more recently, stimulant overdoses [1]. Fentanyl is highly lethal because it powerfully suppresses respiration, leading to potential respiratory failure and death [2]. Its spread into stimulant overdoses involving cocaine and methamphetamines is largely due to the ease with which low-quality batches of these drugs can be adulterated with fentanyl. For drug dealers, fentanyl is appealing due to its low cost and relative simplicity of its manufacture and transport from sources in Mexico and China. Even small fentanyl quantities can yield millions of dollars in sales, particularly with the rising adulteration of illicit stimulants.

The therapeutic challenge arises because the adulteration of cocaine and illicit “Adderall” with fentanyl has incorporated these stimulants into the fentanyl overdose epidemic. This underscores the urgent need for effective therapeutics, particularly blockers, for all three drugs. However, no FDA-approved pharmacotherapies specifically target cocaine, which affects over 5 million abusers; amphetamine, with 3 million abusers; or fentanyl, with 1 million “direct” abusers [3]. The search for therapeutics, particularly blockers, has highlighted the potential of vaccines that produce sustained antibodies (ABs) to neutralize these substances [4]. Despite extensive research over the past 30 years, anti-drug vaccine trials have found that only two-thirds of patients achieve sufficient AB levels to block the targeted drugs. This emphasizes the critical need for biomarkers to not only identify patients likely to respond well, but also to recognize those who may need alternative vaccine formulations to produce the necessary high AB levels.

A completed clinical trial of an anti-cocaine vaccine involving 152 vaccinated patients with stored plasma offers an opportunity to discover biomarkers. Plasma microRNAs (miRs), which have been associated with high-AB responses to various vaccines [5], are promising candidates. Therefore, we designed this post hoc study to replicate these miR studies by comparing the highest- to lowest-antibody responders from the completed cocaine vaccine trial.

The efficacy of vaccines for producing optimal AB levels and affinities varies among individuals and is often enhanced by the inclusion of adjuvants [6]. The vaccine’s antigen is often a bacterial protein or inactivated virus, as seen in recent anti-COVID-19 vaccines. For smaller molecular targets, known as haptens, vaccines are developed by attaching the hapten to a larger immunogenic protein, such as tetanus toxoid or a subunit of cholera B toxoid [7]. This haptenated vaccine approach has been used to develop vaccines against various substances of abuse, including opioids (e.g., fentanyl), stimulants (e.g., cocaine, methamphetamine), and nicotine [7,8]. A common adjuvant used in these vaccines is an aluminum salt, which induces pro-inflammatory cytokine expression and the activation of dendritic cells and macrophages. These cells lead to the priming of naïve T cells and provoke antigen-specific immune responses, including B cell activation and AB production [6].

Chronic inflammation can impair vaccine efficacy through several mechanisms, including the excessive TNF-alpha suppression of T and B cells [9,10]. Substance use disorder patients develop chronic inflammation from excessive release of neurotransmitters due to the drugs binding to receptors expressed on glial cells and amplifying inflammatory signaling via release of cytokines and chemokines, potentially contributing to the positive feedback that promotes inflammation [11]. The suppression of this chronic inflammation can improve vaccine responses. Innate immune responses are critical to the humoral response for vaccine efficacy, and several miRs regulate and enhance these responses through cytokines suppressing pro-inflammatory expression [5,12,13,14,15,16,,17,18,19]. Thus, miRs are pivotal in regulating vaccine responses and may be particularly important for individuals with active substance use disorders, as chronic inflammation can hinder their AB responses to haptenated vaccines.

Importantly, while treatments to inhibit this response through specific miRs are a future possibility, miRs can more immediately serve as biomarkers for identifying poor or enhanced responders to anti-addiction vaccines, paralleling their emerging roles in cancer, diabetes, Alzheimer′s disease, allergic inflammatory disease, rheumatoid arthritis, and other chronic inflammatory and degenerative diseases [20]. Several studies have recognized the importance of identifying pre-vaccination miR associated with strong AB responses among infectious diseases such as influenza, Ebola, and COVID-19 [5,21,22]. Both whole blood and exosomal miRs were primarily positively correlated with AB levels obtained from 14 to 360 days after vaccination in a small sample of subjects given Ebola vaccination. For Ebola, these included miR-5196, 508, 3179, 5685, 155, and 382 and somewhat different and more diverse miRs for exosomes including 486, 410, 98, 4475, 4763, 1271, 6750, 1973, 3156, 487a, 369, 200a, 372, and 584n [22]. MiR-486, 410, and 98 were particularly interesting for the Ebola study because of their regulation of cytokine response via the NF-kB pathway and IL-6 signaling [23,24]. The influenza vaccine work in 53 older subjects found a more consolidated group of three, miR-150, 629, and 4443, that were correlated with immune outcomes across several cell types particularly in CD4+ T cells [21]. Important signaling pathways included TGF-beta, PI3K-Akt, p53, MAPK, TNF, and C-type lectin receptor signaling. Earlier influenza vaccine studies found miR-451 and miR-192 to be important for AB responses, but these were not important for COVID vaccination [25]. The COVID vaccine study using Pfizer BNT162b2 in 61 subjects focused on sixteen exosomal miRs in blood and identified two of interest, miR92a-2-5p and 148a, and three others with weaker associations with AB levels: miR-132, 221, and 625-3p [5]. Thus, the aim of this post hoc study is to replicate previous findings on miR biomarkers for predicting strong AB responses with anti-addiction vaccines. This study compares two subgroups in the completed cocaine vaccine clinical trial: the highest- and lowest-AB responders.

## 2. Materials and Methods

### 2.1. Subjects and Vaccination Procedures

Our team enrolled 300 patients into a randomized, placebo-controlled clinical trial of the anti-cocaine vaccine, TA-CD, from 2010 to 2012 (ClinicalTrials.gov, NCT00969878). The TA-CD vaccine consists of succinyl norcocaine covalently linked to cholera toxin B (SNC-rCTB), adsorbed onto an aluminum hydroxide adjuvant. Subjects were randomized to five 0.5 mL intramuscular vaccinations of 400 micrograms of active (SNC-rCTB) vaccine at weeks 1, 3, 5, 9, and 13 [26]. Due to a higher completion rate among the high-AB-response group compared to the low group (93% vs. 80%), we focused our post hoc analysis on participants who completed at least 3 injections and had AB levels assessed at week 9, which was 4 weeks after the second booster. We selected stored frozen plasma from 48 participants for comparison, comprising 25 with the highest IgG AB responses and 23 with the lowest responses among the 152 actively vaccinated participants [27].

Blood samples were collected prior to the first vaccination, and no participants had urine samples indicating recent cocaine use. All participants met DSM-IV criteria for current cocaine use disorder, having provided a benzoylecgonine-positive urine sample within two weeks prior to blood sampling and study randomization. As expected, mean IgG AB levels at week 9 significantly differed between the two groups (16.7 vs. 132.0 mcg/mL; *p* < 0.01). As previously published, the cholera B toxoid-based vaccine produced significant IgG AB levels against this carrier protein in all patients, supporting the specificity of variations in the anti-cocaine AB as independent of the response to the cholera carrier [26,27]. Interestingly, IgM and IgA AB levels were not correlated with IgG AB responses, and pre-vaccination IgM levels against cocaine predicted poor anti-cocaine IgG levels after vaccination [27,28].

Baseline drug and alcohol use over the past month for these patients included a mean of 14 days (range: 7–22) for cocaine, 0 days (0–2) for cannabis, 4 days (1–15) for alcohol, and 0 days (0–4) for alcohol intoxication. Lifetime cocaine use averaged 16 years (range: 10–22). The two groups did not differ in any of these drug use characteristics. Additionally, the demographics of the AB response groups were similar, with 19 out of 25 versus 17 out of 23 males, 15 out of 25 versus 15 out of 23 whites, and mean ages of 45.5 versus 44.3 years for the highest- and lowest-AB response groups, respectively.

### 2.2. Epigenetic microRNA Procedures

The RNA was extracted from 250 μL serum samples using the miRNeasy Serum/Plasma Kit (Qiagen Cat: 217184). miRNA libraries were then prepared with the QIAseq miRNA Library Kit (Qiagen, Germantown, ML, USA, Cat: 331505). The prepared libraries were pooled at 2 nm and sequenced at the University of Houston UH-Seq Core using an Illumina NextSeq 2000, generating 10 million 76bp reads per sample. We processed the miR-Seq data using the ENCODE pipeline, performing mapping with STAR [29] and miRNA quantification using the miRBase reference [30]. In addition to whole blood plasma miR, we also extracted exosomes from the blood and sequenced those miRs separately for a parallel analysis. Finally, we and others have performed repeated miR sequencing on samples stored at −80 °C for months and years and found that the batch effect changes across these time intervals were minimal [31].

### 2.3. Statistical Analyses

Plasma DE miRs were compared between the high- and low-AB groups by analyzing the differential expression of each miR and adjusting significance levels for multiple comparisons using an FDR control. These two-way comparisons were displayed using volcano plots separating up-regulated from down-regulated DE miRs at the significance threshold of 2-fold DE and FDR adjusted *p*-value < 0.05. We also conducted sensitivity analyses by adjusting the FDR to <0.001 and considering DE miRs with 4- to 16-fold differences, allowing us to assess how the number of significant DE miRs were affected by these more stringent thresholds. The low versus high classification of patients before vaccination were compared for accuracy of that classification using a Random Forest technique. Cross-validation used 100 iterations, with samples split into 70% training and 30% testing sets. Since we expected to find about 50 DE miRs, we anticipated many gene targets, which we determined using miRDB [32].

We then reduced the number of essential miRs for identifying participants with optimal vaccine AB responses by increasing the FDR significance from *p* < 0.05 to *p* < 0.001 for the mean differences in individual miRs and by finding multiple miRs that converged on individual genes. In the volcano plot, the fold change is expressed as log_2_ (e.g., 1 = 2-fold; 2 = 4-fold; 3 = 8-fold) and the significance levels (*p* < 0.00) for FDR values are expressed as −log_10_ (e.g., 1.3 = 0.05, 2 = 0.01, 3 = 0.001).

## 3. Results

**Figure 1:**
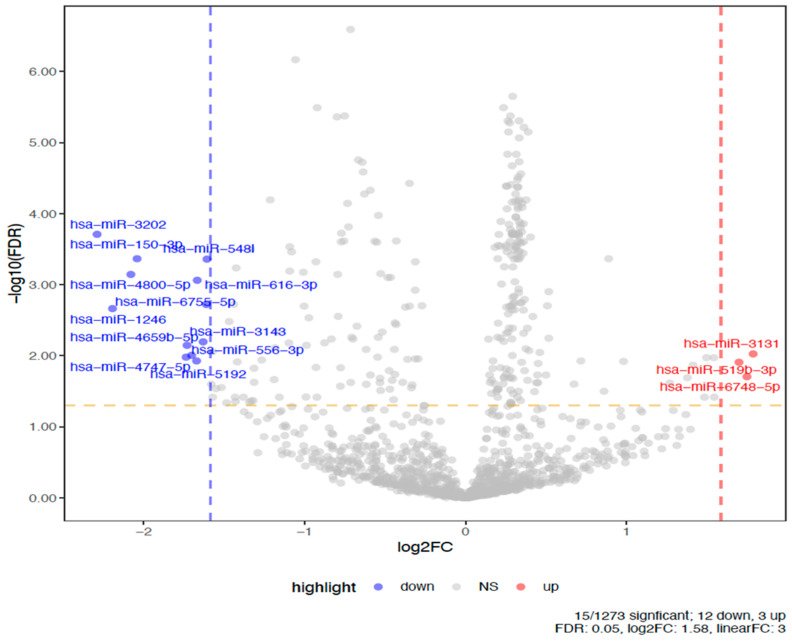
Plot of mean DE-miR for subjects with high vs. low cocaine AB levels. (Significantly different miRs (FDR *p* < 0.05 and fold change (FC) > 3-fold) between subjects having high versus low peak AB levels are labeled and in two colors—blue or red. Blue dots are significantly down-regulated miRs and red dots are significantly up-regulated miRs; gray dots are miRs that show no significant difference).

**Figure 2:**
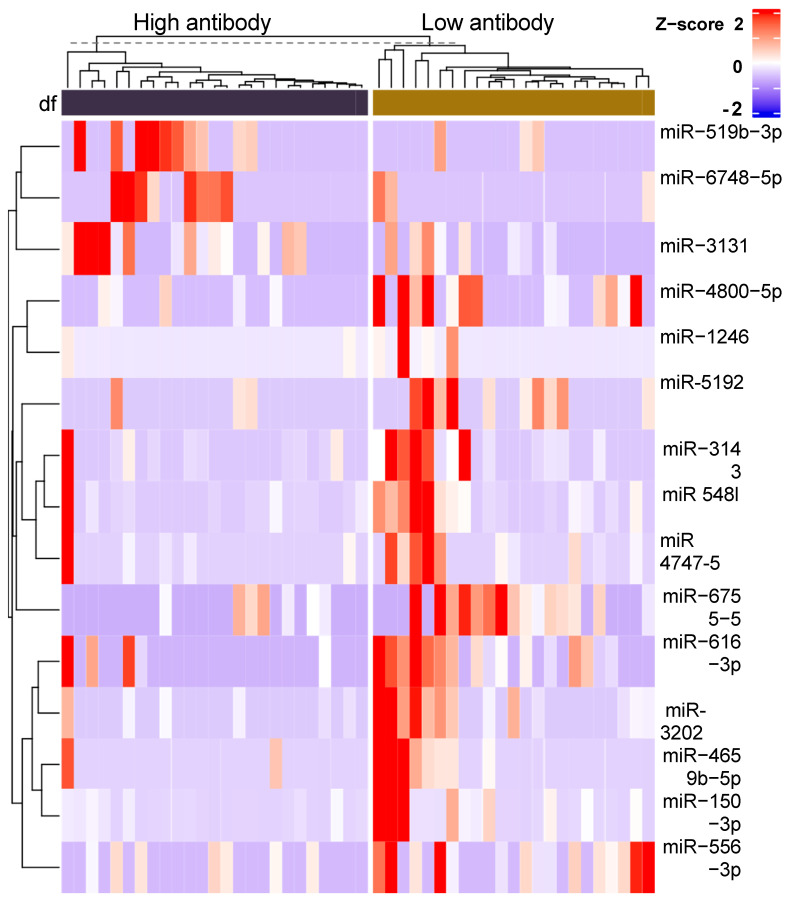
Heatmap of individual subject’s AB level Z-scores for every microRNA that significantly distinguished subjects in high- versus low-AB groups.

Figure 1 is a volcano plot showing the 2517 differentially expressed (DE) plasma miRs included in the comparison between the high- and low-AB-level responders. For the low- compared to high-AB responders, the right upper corner shows the up-regulated DE-miRs, while the upper left corner shows the down-regulated DE-miRs. Using a DE-FDR < 0.05 and a >3-fold difference between responders yielded 12 miRs that were down-regulated and 3 miRs that were up-regulated compared to low-AB patients. These 15 miRs are labeled in Figure 1. The three up-regulated miRNAs were miR-3131, -519b, and -6748. The 12 down-regulated miRNAs were miR-3202, -150, -5481, -4800, -616, -6756, -1246, -4659, -3143, -556, -4747, and -5192. Figure 2 provides a heatmap showing the contrasting up- or down-regulation for each of the DE-miR for the high- versus low-AB responders. A similar analysis using the exosomal miR yielded 12 DE-miRs that differed > 3-fold at an FDR < 0.05. The exosomal miRs were miR-106b, 140, 142, 146a, 1273g, 32, 320c, 423, 4516, 4532, 6087, and 663.

**Figure 3:**
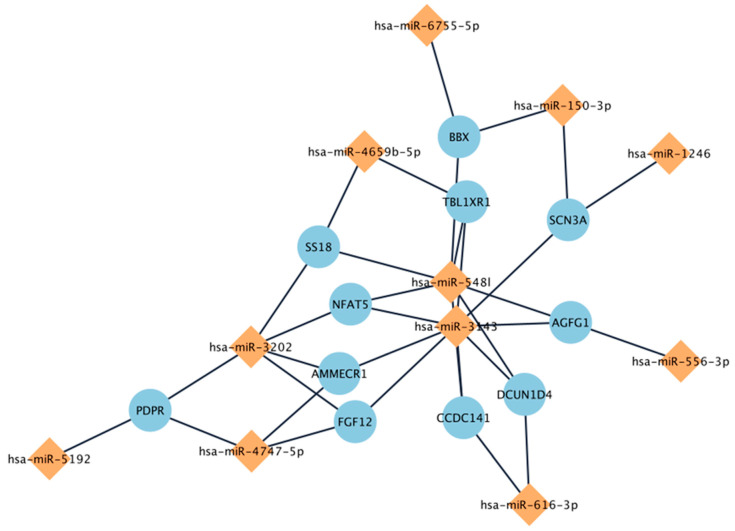
Genes targeted by three or more of eleven significantly down-regulated miRs (blue circles are genes, and orange diamonds are miRs with lines showing genes regulated by a particular miR).

The 3 up-regulated miRs targeted 382 unique genes, while the 12 down-regulated miRs targeted 1673 genes. None of the three up-regulated miR genes targeted overlapping genes, while three or more of the twelve down-regulated miRs targeted 11 genes as shown in Figure 3. Figure 3 also shows three down-regulated miRs targeting four to seven different genes: miR-3202, -5481, and -3243. The potential relevance of these 12 down-regulated miRs to immune function was also highlighted by a tissue-type enrichment analysis of the targeted genes showing that gastric immune cell genes showed the strongest association of all the tissues analyzed (*p* < 10^−8^) [33].

## 4. Discussion

Comparing patients with high versus low IgG AB levels induced by a cholera-based anti-cocaine vaccine, we found that 15 out of 2517 plasma whole blood miRs distinguished these two groups (FDR *p* < 0.05), each exhibiting at least a three-fold difference in expression. In total, 12 of these 15 miRs were down-regulated in the high- versus low-AB groups, and the 12 miRs targeted 1673 genes. However, focusing on genes targeted by three or more of these miRs yielded only 11 genes. Furthermore, three miRs targeted four to seven different genes: miR-3202, -5481, and -3243. Thus, as few as three down-regulated miRs or as many as 15 miRs may be the strongest candidates for consideration as biomarkers for high-AB responders to an anti-drug vaccine. A parallel analysis of the exosomal miR yielded only 12 miRs at these same DE parameters, and none of these miRs overlapped between the whole blood and exosomal extractions. This lack of overlap in correlations of post-vaccination AB levels and pre-vaccination miR profiles from exosomal compared to whole blood miR was also described with Ebola virus vaccine [22]. That Ebola study identified significant correlations of specific miR with AB levels at 4 and 8 weeks after vaccination. None of those nine strongest correlations of whole blood miR with AB levels overlapped with the current study’s 15 miRs shown in Figure 2, although both studies had relatively small sample sizes of 48 currently and 30 for Ebola.

An alternative approach to finding the most important DE-miRs for distinguishing high-AB responders uses the gene targets of these DE-miRs, as applied through tissue-specific enrichment [33]. This enrichment found that gastric immune cell genes showed the strongest association of all the tissues analyzed (*p* < 10^−8^). A similar GAD-disease class enrichment yielded a very strong association with infections (*p* < 0.0003) [34]. These tissue- and disease-specific associations likely reflect our use of cholera toxoid B as the cocaine hapten carrier for the anti-cocaine vaccine, leading to shared genes for AB production between this anti-cocaine vaccine and the cholera infectious disease vaccine (7). The specificity for gastric immune cell pathways may further reflect cholera’s induction of diarrhea as its critical gut toxicity and symptom. Using a third enrichment database—the GOTERM BP Direct enrichment—we found that messenger RNA translation and stability had particularly strong associations with our DE miRs (*p* < 10^−5^ to <10^−9^) [33]. Thus, the DE miRs found in our vaccine study appear to be relevant to the formation of AB against cholera, and the patients with the greater AB production also appear to have more effective pathways for specifically making miRs that inhibit messenger RNA translation.

The association of specific miRs with immunity and vaccine AB responses has implicated several miRs that regulate inflammation and cell proliferation in B cells [33,34,35,36,37,38].

Besides the Ebola vaccine, vaccination against COVID-19 has examined miRs as predictors of AB responses. The COVID vaccine study using Pfizer BNT162b2 in 61 subjects assessed only sixteen exosomal miRs in blood and identified two of interest, miR-92a-2-5p and 148a, and three others with weaker associations to AB levels: miR-132, 221, and 625-3p [5,25]. We did not identify any of these five miRs among those important for AB levels after our anti-cocaine cholera-based vaccination. However, we examined a much larger set of about 2650 miRs in both whole blood and exosomes for associations with AB levels in a similar-size sample of 48 subjects selected for high- versus low-AB responses within a sample of about 150 subjects.

Another important vaccine has been against influenza, and early vaccine studies found miR-451, miR-192, and miR-150 to be important for AB responses, and specifically elevated serum levels of miR-150 were found following MF-59-adjuvanted 2009 influenza A (H1N1) vaccination in subjects with higher AB titers [39]. Since the overexpression of miR-150 impairs the formation of mature B cells and their functional activity of making AB, reducing the expression of miR-150 could enhance the AB production following vaccination [40,41]. Interestingly, a more recent influenza study in 53 older subjects found that miR-150 was correlated with immune outcomes across several cell types, particularly in CD4+ T cells [21]. Important signaling pathways included TGF-beta, PI3K-Akt, p53, MAPK, TNF, and C-type lectin receptor signaling. We similarly found that miR-150 was the second most significant DE-miR distinguishing the high- versus low-AB responders. An additional recent study found that increased miR-150 expression in B cells was negatively correlated (*p* = 0.00038) with memory B cell ELISPOT response after influenza vaccine, likely due to mature B cell suppression or the suppression of germinal center memory B cell differentiation [20,39,41]. As further support, they found increased miR-150 expression in T cells (CD4+ and CD8+ T cells) that was negatively correlated with the AB titer. Interestingly, the intracellular miR-150 expression in T cells (e.g., CD4+ T helper cells) is down-regulated upon T cell activation to control key genes underlying T cell functions and the miR-150 is quickly released extracellularly and can be detected in blood [39,42].]. Extracellular miR-150 levels have also been a reliable serum marker of lymphocyte activation (T cell activation) [43]. Thus, miR-150 expression appears to be a reliable biomarker of influenza vaccine-induced immunity in two previous studies [33,34] as well as our current study.

## 5. Conclusions and Limitations

The findings of this post hoc study underscore the potential of specific miRs as biomarkers for predicting AB responses to the anti-cocaine vaccine. Our study is limited by several factors. First, its modest sample size of 48 subjects will need replication in a larger sample with a broader range of AB levels. Second, the unique focus on a haptenated vaccine that critically depends on its antigenic carrier protein cholera B toxoid for developing an antibody response is different from the infectious disease vaccines previously studied. Since our toxoid carrier showed no significant variation across subjects in its anti-cholera AB levels, our findings may not be directly relevant to infectious disease variations in AB response. Third, we focused on IgG levels, but our recent analyses suggest that IgA may be a more important component for the therapeutic effect of our vaccine for blocking cocaine abuse and may merit further examination [28]. Fourth, examining miR associated with the AB responses after the initial and three booster vaccinations may provide critical insight into how to improve this type of anti-drug vaccine efficacy. Fifth, examining all 2650 miRs reduced our statistical power compared to selecting more focused groups of miR, as had been carried out by previous studies with infectious diseases. Sixth, focusing on exosomal miR rather than whole blood miR would yield different results for our hub gene and related analyses, but using whole blood miR enabled a more detailed analysis of a larger group of miR than we found with exosomal miR. Seventh, we did not examine specific B cell functional impacts from the miR, and future studies will need to address this mediator type of an analysis. Ultimately, this study has limited generalization to other vaccines, because anti-addiction vaccines are for a special population who will also need better vaccines to address the low anti-cocaine AB levels in some participants.

Overall, replicating DE-miRs from two previous vaccine studies demonstrate the potential to identify additional replicated miRs among the other 14 significant miRs associated with high AB levels. Additionally, 3 of the 12 down-regulated DE-miRs (miR-3202, -5481, -3243) appear to jointly target four to seven genes, suggesting that these gene products may be particularly important for inhibiting AB responses to this vaccine. Overall, our three approaches to identifying the optimal miR for selecting patients most likely to benefit from these anti-addiction vaccines can be used sequentially, as we carried out. First, we found those DE-miRs that individually separate the high- from low-AB responders. Second, we selected those DE-miRs that act synergistically on genes and third, we used disease- or tissue-specific enrichments with hub genes that focus on relevant disorders like infectious disease and on processes like messenger RNA translation, which miRs specifically regulate and disrupt. Thus, the range of genes regulated by miRs can be found and provide valuable insights into the biological mechanisms underlying vaccine efficacy and become potential miR biomarkers for improving vaccination outcomes.

## Data Availability

Data supporting reported results can be obtained through the first author, TRK, for use in appropriately approved research protocols that are consistent with the stated aims of this secondary analysis of the originally collected data from IRB protocol H-23257 funded by NIDA and conducted by TRK as PI.
